# Is the Dental Profession Crowded?

**Published:** 1898-03

**Authors:** 


### Article IX.

### IS THE DENTAL PROFESSION CROWDED?

This is a question often presented in private conversation between dentists, and sometimes at dental meetings. That the profession is crowded hardly needs to be affirmed. Only a few more years and every nook and cranny of the country will be jammed as are now the towns and cities.

From Canada comes the word that they are overflowing and still they come. The dental statistician will say that a certain per cent. of the older practitioners die every year; and that a certain per cent. of the recruits practice only a little while and quit. And yet there is a large increase in the ranks every year. The State of Georgia added 18 per cent. last year. The over crowding of any business sharpens competition. Sharp competition tends to lower prices.

The professions are not exceptions to this rule; certainly the dental profession is not, for a large part of the world does not yet look upon it as one of the learned professions. And will they do it so long as teeth are held in the jaws by gomphosis, and can be removed without physical disability following?

A dentist's standing is rated by the public by the "sticking of his fillings." His scientific attainments go for naught if his mechanical ability is not sufficient to make fillings "stick." These truths may be humiliating, but they are truths nevertheless, and tend to prove that it is easier to overcrowd the dental profession than that of medicine. though it is almost crowded to suffocation.

The cutting and slashing of prices tend to lessen professional enthusiasm and ambition, and if carried far enough, result in charlatanry.

Is this pessimistic? We think not. One only needs to turn to the advertising pages of the journals to see the number of dentists who are now supplementing their living by the manufacture of articles for sale. We are not alluding to the classes, but to the masses. Often have we heard Dr. Crawford, on the floor of convention halls, declare, "There are not enough dentists to keep the tartar off people's teeth." That is, provided the people want the tartar taken from their teeth. As our Canadian correspondent writes: "The question of overcrowding is usually switched off to educating the people up to a sense of dental appreciation." *When* they are so educated, then there may be room for the annual 18 per cent increase. Five years' output at that rate will double the number of dentists. Can any one predict other than a mere existence for the mass of dentists? We are told that the present average earnings of the profession in Chicago is about $1,260; which is not the average salary of first-class salesmen.

We shall have to quote Dr. Crawford again, as he stands as an ex-professor, and has been twice president of the American Dental Association. Not long since he made remarks which do not exactly conform to those quoted above, but which bear out our views exactly. He said, in speaking on the subject of dental education: "The student ought to remain longer in college. His future occupation offers a barrier to all subsequent intellectual development." Why? "Because he is going to have the hardest work to make a living. The most of those that I know are poor."

Not enough dentists to keep the tartar off people's teeth, and yet the young dentist will have such a struggle to make a living, he will not develop mentally. 'Tis a sad fact that most of them don't grow intellectually, but are content to remain as they started in professional life.

When a condition exists, there must be a reason for its existence.—*The American Dental Weekly.*

---

ARTICLE X.

